# Study on the absolute configuration and biological activity of rotenoids from the leaves and twigs of *Millettia pyrrhocarpa* Mattapha, Forest & Hawkins, sp. Nov

**DOI:** 10.1186/s12906-023-03963-4

**Published:** 2023-05-04

**Authors:** Suda Sananboonudom, Atchara Kaewnoi, Wilart Pompimon, Samroeng Narakaew, Suwadee Jiajaroen, Kittipong Chainok, Narong nuntasaen, Kanoknetr Suksen, Arthit Chairoungdua, Jitra Limthongkul, Chanita Naparswad, Suttiporn Pikulthong, Puttinan Meepowpan, Boonthawan Wingwon, Nichapa Charoenphakinrattana, Phansuang Udomputtimekakul

**Affiliations:** 1 Department of Chemistry, Faculty of Science and Center of Innovation in Chemistry, Lampang Rajabhat University, Lampang, 52100 Thailand; 2 Department of Thai Traditionnal Medicine, Faculty of Science and Technology, Bansomdejchaopraya Rajabhat University, Bangkok, 10600 Thailand; 3 Thammasat University Research Unit in Multifunctional Crystalline Materials and Applications (TUMcMa), Faculty of Science and Technology, Thammasat University, Pathum Thani, 12121 Thailand; 4 Department of Chemistry, Faculty of Science and Center of Innovation in Chemistry, Mahidol University, Bangkok, 10400 Thailand; 5 Department of Physiology, Faculty of Science, Mahidol University, Bangkok, 10600 Thailand; 6 Excellent Center for Drug Discovery (ECDD), Mahidol University, Bangkok, 10600 Thailand; 7 Toxicology Graduate Program, Faculty of Science, Mahidol University, Bangkok, 10600 Thailand; 8 Department of Microbiology, Faculty of Science, Mahidol University, Bangkok, 10600 Thailand; 9 Department of Chemistry, Faculty of Science, Mahidol University, Bangkok, 10600 Thailand; 10 Department of Chemistry, and Center for Innovation in Chemistry, Faculty of Science, Chiang Mai University, Chiang Mai, 50300 Thailand; 11 Department of Management Science, Faculty of Management Science, Lampang Rajabhat University, Lampang, 52100 Thailand; 12 Life Group International, Khaochangum Subdistrict, Photharam District, Ratchaburi, 70120 Thailand

**Keywords:** *Millettia pyrrhocarpa*, Fabaceae: Faboideae, Rotenoids, Antibacterial activity, Anti-HIV activity, Cytotoxicity

## Abstract

**Background:**

*M. pyrrhocarpa* is a new plant in the Fabaceae: Faboideae family that is found in Thailand. A literature search revealed that the *Milletia* genus is rich in bioactive compounds possessing a wide range of biological activities. In this study, we aimed to isolate novel bioactive compounds and to study their bioactivities.

**Methods:**

The hexane, ethyl acetate, and methanol extracts from the leaves and twigs of *M. pyrrhocarpa* were isolated and purified using chromatography techniques. These extracts and pure compounds were tested in vitro for their inhibitory activities against nine strains of bacteria, as well as their anti-HIV-1 virus activity and cytotoxicity against eight cancer cell lines.

**Results:**

Three rotenoids, named 6a*S*, 12a*S*, 12*S*-elliptinol (1), 6a*S*, 12a*S*, 12*S*-munduserol (2), dehydromunduserone (3), and crude extracts were evaluated for antibacterial, anti-HIV, and cytotoxic activities. It was found that compounds 1–3 inhibited the growth of nine strains of bacteria, and the best MIC/MBC values were obtained at 3/ > 3 mg/mL. The hexane extract showed anti-HIV-1 RT with the highest %inhibition at 81.27 at 200 mg/mL, while 6a*S*, 12a*S*, 12*S*-elliptinol (1) reduced syncytium formation in 1A2 cells with a maximum EC_50_ value of 4.48 μM. Furthermore, 6a*S*, 12a*S*, 12*S*-elliptinol (1) showed cytotoxicity against A549 and Hep G2 cells with maximum ED_50_ values of 2.27 and 3.94 μg/mL.

**Conclusion:**

This study led to the isolation of constituents with potential for medicinal application, providing compounds (1–3) as lead compounds against nine strains of bacteria. The hexane extract showed the highest %inhibition of HIV-1 virus, Compound 1 showed the best EC_50_ in reducing syncytium formation in 1A2 cells, and it also showed the best ED_50_ against human lung adenocarcinoma (A549) and human hepatocellular carcinoma (Hep G2). The isolated compounds from *M. pyrrhocarpa* offered significant potential for future medicinal application studies.

**Supplementary Information:**

The online version contains supplementary material available at 10.1186/s12906-023-03963-4.

## Background

*Millettia pyrrhocarpa* Mattapha, Forest & Hawkins, sp. Nov. belongs to the family Fabaceae: Faboideae and is found in Nakorn Nayok province, Thailand. *M. pyrrhocarpa* is a woody climber plant that is known in Thai as “Nang Rong” [1]. Previous phytochemical analyses of the *Milletia* genus revealed that various secondary metabolites were present, such as alkaloids [2], coumarins [3], flavonoids [4], isoflavonoids [5], phenols [6], phytosterols [2], rotenoids [7–10], and triglycerides [11]. Among these, rotenoids are the most common in this genus [12], as observed in the leaves of *Millettia oblate* ssp. *teitensis* [13], *Millettia brandisiana* KURZ [14] and in the root bark of *Millettia usaramensis* [15] and *Millettia speciosa* [16]. Pharmacological investigation of the *Millettia* genus revealed that the crude extract and isolated compounds showed antimicrobial [2, 17, 18], antioxidant [2, 18], antiplasmodial [7, 10], immunomodulatory [19], anti-cholinesterase [20, 21], anthelmintic [22], anti-inflammatory [5, 23], antidiabetic [24], cytotoxic [6, 16, 20], and anticancer [6, 20, 25] activities. Since the phytochemical study and biological activity of *M. pyyrhocarpa* has not been reported, it is of great interest to study the secondary metabolites in the leaves and twigs of *M. pyyrhocarpa* and their antibacterial, anti-HIV1-RT, and cytotoxic activities. The preliminary evaluation of the bioactivity of the leaves and twigs of *M. pyyrhocarpa* might lead to the discovery of important substances that exhibit promising biological activity and potential to be developed for medicinal applications in the future.

## Material

### Plant material

Leaves and twigs of *Millettia pyrrhocarpa* were collected from Nakhon Nayok Province, Mueang district, Hin Tang subdistrict, Khao Yai National Park, Nang Rong waterfall in Thailand and identified by Dr. Narong Nantasean. Voucher specimens of *M. pyrrhocarpa* (*M. pyrrhocarpa* Mattapan, & BKF staff 1139B holotype BKF) were deposited at the Department of Chemistry, Faculty of Science and Center of Innovation in Chemistry, Mahidol University, Rama VI Road, Bangkok 10400, Thailand.

Cells line materials KKU-M213 (human cholangiocarcinoma) cells were kindly provided by Dr. Banchob Sripa from Liver Fluke and Cholangiocarcinoma Research Center, Department of Pathology, Faculty of Medicine, Khon Kean University. MMNK1 (human cholangiocyte) cells were obtained from the Japanese Collection of Research Bioresources Cell Bank (JCRB, Osaka, Japan). FaDu (human hypopharyngeal carcinoma), HT29 (human colorectal adenocarcinoma), MDA-MB-231 (human mammary gland/breast adenocarcinoma), SH-SY5Y (human neuroblastoma), A549 (human lung carcinoma), and HepG2 (human hepatocellular carcinoma) cell lines were obtained from the American Type Culture Collection (ATCC, Manassas, VA, USA).

## Methods

### General experimental procedures

Column chromatography (CC) was carried out on silica gel 60 H from E. Merck. 70–230 mesh ASTM, cat. No. 7734 and No.7736. Thin-layer chromatography (TLC) separations were carried out on silica gel 60 PF_254_ on aluminium sheets, and the isolated compounds were identified under ultraviolet light. Infrared spectra (IR) were recorded as KBr pellets using a Shimadzu 8900 FT-IR spectrophotometer. Melting points were recorded on a Büchi 322 micro melting point apparatus and are uncorrected. Mass spectra were recorded on a Thermo Finnigan Polaris Q mass spectrometer at 70 eV (probe), and EIMS was measured by a Brüker Esquire apparatus. X-ray absorption spectroscopy was carried out on a Bruker D8 QUEST CMOS PHOTON II. ^1^H (500 MHz), ^13^C (125 MHz), and 2D NMR spectra were recorded on a Bruker AV-500 spectrometer in deuterated chloroform (CDCl_3_) or deuterated methanol (CD_3_OD) solutions, and TMS was used as the internal standard.

### Extraction and isolation

Air-dried powders of the leaves and twigs of *M. pyrrhocarpa* (2.8 kg) were extracted successively with hexane, EtOAc, and MeOH. Twelve litres of solvent were used in the extraction at room temperature for three days in triplicate. The solvents were filtered and evaporated under reduced pressure to give crude hexane (41.0 g), EtOAc (118.0 g), and MeOH (216.0 g) extracts. The crude hexane extract was fractionated using silica gel column chromatography (CC) using a gradient system of hexane–EtOAc and EtOAc–MeOH to generate ten fractions (A1-A10). Fraction A5 (13.6 g) was fractionated on a silica gel column and eluted with a gradient system of hexane–EtOAc and EtOAc–MeOH to give three subfractions (B1-B3). Subfraction B2 (5.1 g) was fractionated on a silica gel column and eluted with a gradient system of hexane–ethyl acetate and EtOAc–MeOH to give four subfractions, C1-C4. Subfraction C1 (0.06 g) was recrystallized with ethanol:ethyl acetate (2:1) to give compound (1) (70 mg) as a white solid. Subfraction C3 (0.41 g) was recrystallized with ethanol:ethyl acetate (2:1) to afford compound (3) (40 mg) as a white solid. The crude EtOAc extract (118.0 g) was fractionated on a silica gel column and eluted with a gradient system of hexane–EtOAc and EtOAc–MeOH to afford nine fractions (D1-D9). Fraction D2 (23.3 g) was fractionated on a silica gel column and eluted with a gradient system of hexane–ethyl acetate and EtOAc–MeOH to produce seven subfractions (E1-E7). Subfraction E4 (3.0 g) was recrystallized with ethanol:ethyl acetate (2:1) to generate compound (1) (86 mg) as a white solid. The crude MeOH extract (216.1 g) was fractionated on a silica gel column and eluted with a gradient system of hexane–EtOAc and EtOAc–MeOH to generate nine fractions (G1-G9). Fraction G3 (1.3 g) was recrystallized with ethanol to afford compound (2) (36 mg) as a white solid.

### X-ray diffraction

The colourless plate of compound (2) was suitable for single-crystal X-ray diffraction with a size of 0.32 × 0.28 × 0.04 mm. The unit cell parameters and intensity data were recorded on a Bruker D8 QUEST CMOS PHOTON II diffractometer equipped with a graphite-monochromator Mo-Kα (λ = 071,073 Å) radiation at 296(2) K. Data reduction was performed using SAINT, and the SADABS-2016/2 scaling algorithm [26] was used for absorption correction. The structure was solved with the ShelXT structure solution program using combined Patterson and dual-space recycling methods [27]. The structure was refined by least squares using ShelXL [28]. All non-H atoms were refined anisotropically. The hydrogen atoms of solvent molecules were positioned geometrically with C—H = 0.93–0.98 Å and refined using a rigid model with fixed displacement parameters *U*_iso_ (H) = 1.5*U*_eq_ (C) for methyl groups and 1.2*U*_eq_ (C) for the other groups. The O–H hydrogen atoms were located on difference Fourier maps but refined with O–H = 0.82 ± 0.02 Å with *U*_iso_ (H) = 1.5*U*_eq_ (O). C_19_H_20_O_6_, FW = 344.35, orthorhombic space Group *P*_21_*P*_21_*P*_21_, unit cell dimensions *a* = 6.40000(10) Å, *b* = 9.9684(2) Å, *c* = 25.9394(6) Å, *V* = 1654.88(6) Å^3^, *Z* = 4, *d*_calcd_ = 1.382 g/cm^3^, *µ* = 0.103 mm^−1^, *F*(000) = 728. The 46671 measurements yielded 6568 independent reflections after equivalent data were averaged. The final refinement gave *R*_1_ = 0.0393 and *wR*_2_ = 0.0896 [*I* > 2σ(*I*)]. The crystallographic data of the compound have been deposited in the Crystallographic Open Database (COD) number 3000415. The molecular graphic was illustrated by ORTEP [29].

## Antibacterial activity

### Bacterial strains

The study on in vitro antibacterial activity was carried out against nine strains (*S. aureus* ATCC 25923 DMST 8840, *E. aerogenes* ATCC13048 DMST 8841, *E. coli* O157: H7 DMST 12743, *E. coli* Enterotoxigenic, ETEC DMST 30543, *E. coli* Enteropathogenic, EPEC DMST 30546, *S. typhimurium* ATCC 13311 DMST 562, *S. flexneri* DMST 4423, *P. mirabilis* DMST 8212, and *V. cholera* nonO1/nonO139 DMST 2873). The minimum inhibitory concentration (MIC) and minimum bactericidal concentration (MBC) of the crude extracts and isolated compounds were evaluated according to standard methods described in previous literature [30].

### Anti-HIV1-RT and cell-based assay for anti-HIV-1

Anti-HIV1-RT assays of the crude extracts and isolated compounds were evaluated according to the standard methods described in previous literature [31–33]. Cell-based assays for anti-HIV-1 RT of the crude extracts and isolated compounds were evaluated according to the standard methods described in previous literature [34, 35]

### Cytotoxicity assay

Cytotoxicity assays of the crude extracts and isolated compounds were evaluated according to the standard methods described in previous literature [36, 37].

## Results

### Isolation and purification

The crude hexane, ethyl acetate, and methanol extracts of the leaves and twigs of *M. pyrrhocarpa* were subjected to repeated chromatography over silica gel 60 and silica gel 60 PF_254_ to yield three pure compounds, (1–3). Compounds (1–3) were identified as elliptinol (1) [38, 39], munduserol (2) [40], and dehydromunduserone (3) [40] by comparison of their spectral data with those in the literature. The ^1^H and ^13^C-NMR data of. The ^1^H and ^13^C-NMR data of (1), (2), and (3) are shown in Table 1.

**Table 1:** ^1^H and ^13^C data for compounds (1–3) (500 MHz, 125 MHz, CDCl_3_ or CD_3_OD, δ in ppm)

**Position**	**6a*S***_**,**_**12a*S***, 12*S***-Elliptinol (1)**		**6a*S***_**,**_**12a*S*, 12*S*-Munduserol (2)**		**Dehydromunduserone (3)**	
	**δ**_**H**_** (*J* in Hz)**	**δ**_**C**_	**δ**_**H**_** (*J* in Hz)**	**δ**_**C**_	**δ**_**H**_** (*J* in Hz)**	**δ**_**C**_
1	7.72, s	111.1	7.75, s	112.1	6.53, s	100.4
2	-	143.9	-	143.6	-	144.0
3	-	149.2	-	149.3	-	149.0
4	6.43, s	100.3	6.44, s	100.5	8.43, s	110.0
4a	-	148.0	-	148.5	-	146.2
5	-	-	-	-	-	-
6	4.58,dd (10.2, 4.3)	67.1	4.42, dd (10.0, 4.3)	66.9	4.99, s	64.8
	4.13, t (10.2)		4.00, t (10.0)			
6a	4.33, dt (10.2, 4.3)	71.8	4.16, td (10.0, 4.3)	71.6	-	156.6
7	-	-	-	-	-	-
7a	-	155.9	-	154.8		156.7
8	-	116.9	6.39, d (2.6)	100.3	6.81, d (2.3)	100.2
9	-	147.5	-	160.3	-	163.9
10	7.21, d (8.1)	105.7	6.60, dd (10.0, 2.6)	107.9	6.98, dd (2.3, 8.9)	114.6
11	7.48, d (8.1)	123.6	7.43, d (10.0)	128.7	8.16, d (8.9)	127.5
11a	-	112.5	-	119.9	-	118.5
12	4.98, t (10.2)	70.7	4.74, d (10.0)	69.7	-	174.3
12a	3.15, t (10.2)	43.7	3.04,t (10.0)	42.3	-	111.9
12b	-	119.8	-	113.4	-	110.5
13	6.83, d (2.1)	103.8	-	-	-	-
14	7.58, d (2.1)	144.5	-	-	-	-
2-OCH_3_	3.89, s	55.8	3.80, s	55.8	3.95, s	56.3
3-OCH_3_	3.85, s	56.4	3.78, s	54.9	3.86, s	55.9
9-OCH_3_	-	-	3.76, s	54.4	3.90, s	55.8
12-OH	1.95, d (10.2)	-	-	-	-	-

6a*S*, 12a*S*, 12*S*-Elliptinol (1): white solid (EtOH:EtOAc); mp = 230 − 231 °C;  ${\left[\alpha \right]}_{589}^{26.9}$ : + 173.36 (*c* 0.38, CHCl_3_); UV (MeOH) λ_max_ (log ε) 235 (1.37), 281 (1.24), 341 (1.09), 447 (1.35), 494 (0.93), 671 (0.91) nm; IR (KBr) υ_max_ 3470, 3102, 2969, 2938, 1620, 1586, 1502, 1273, 1253, 1190, 1142, 1119, 1092, 1041, 989 cm^−1^; for ^1^H and ^13^C NMR spectroscopic data, see Table 1. EIMS m/z 340.31 (base peak), 226.22, 81.05.

6a*S*, 12a*S*, 12*S*-Munduserol (2): white solid (EtOH:EtOAc); mp = 240 − 241 °C; ${\left[\alpha \right]}_{589}^{\text{26.}{2}}$ : + 160.03 (*c* 0.14, MeOH); UV (MeOH) λ_max_ (log ε) 231 (1.73), 450 (1.33), 507 (1.23), 680 (1.19) nm; IR (KBr) υ_max_ 3460, 2918, 2851, 1655, 1637, 1618, 1508, 1292, 1157, 1045 cm^−1^; for ^1^H and ^13^C NMR spectroscopic data, see Table 1. EIMS m/z 345.31 (M + H)^+^, 192.17 (base peak).

Dehydromunduserone (3): white solid (MeOH); mp = 202–204 °C [lit. 41, mp = 210 − 211 °C]; UV (MeOH) λ_max_ (log ε) 231 (1.59), 275 (1.46), 306 (1.36), 356 (1.12), 457 (1.01), 507 (0.92), 677 (0.90) nm; IR (KBr) υ_max_ 3441, 3098, 2999, 2955, 2837, 1761, 1726, 1634, 1601, 1566, 1508, 1452, 1441, 1408, 1290, 1246, 1199, 1163, 1105, 1049, 795 cm^−1^; for ^1^H and ^13^C NMR spectroscopic data, see Table 1. EIMS m/z 354.37, 192.29 (base peak), 179.31.

### Antibacterial activity

**Fig. 1:**
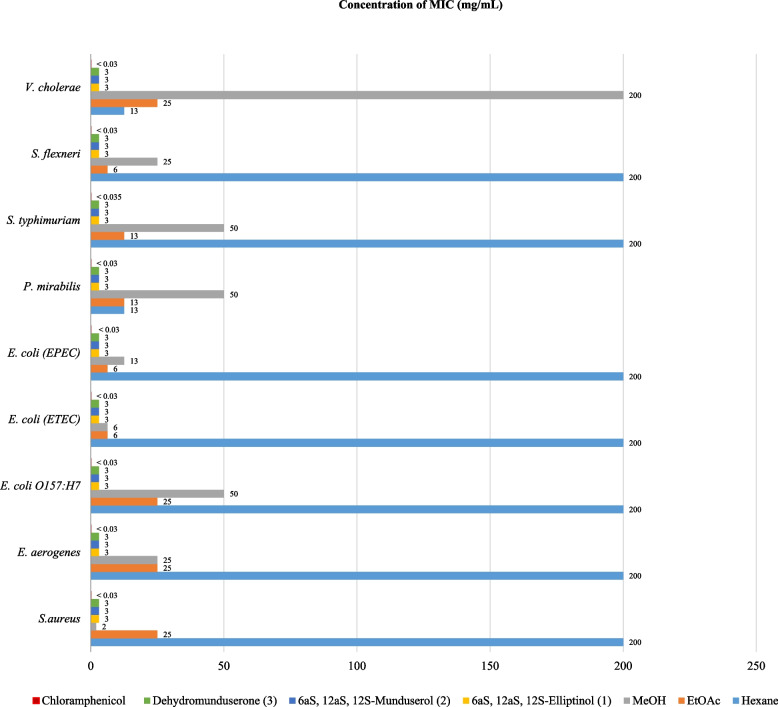
Determination of MIC for crude extracts and pure compounds from leaves and twigs of *M. Pyrrhocarpa*

**Fig. 2:**
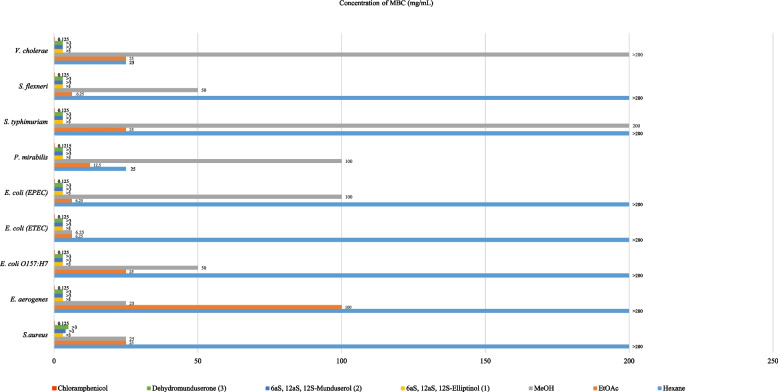
Determination of MBC for crude extracts and pure compounds from leaves and twigs of *M. Pyrrhocarpa*

The three crude extracts and isolated compounds (1–3) were found to inhibit the growth of *V. cholerae* with MIC/MBC values of 12.5/25 mg/mL, while the EtOAc extract inhibited the growth of *E. coli* (ETEC), *E. coli* (EPEC), and *S. flexneri* with MIC/MBC values of 6.25/6.25 mg/mL, and the MeOH extract was found to inhibit the growth of *E. coli* (ETEC) with MIC/MBC values of 6.25/6.25 mg/mL. The isolated compounds (1–3) were found to inhibit the growth of nine bacterial strains with MIC/MBC values of 3 < p3 mg/mL with chloramphenicol as the positive control. These results are shown in Figs. 1 and 2 and were consistent with previous reports [2, 17, 18].

### Anti-HIV-1 RT activity

**Fig. 3:**
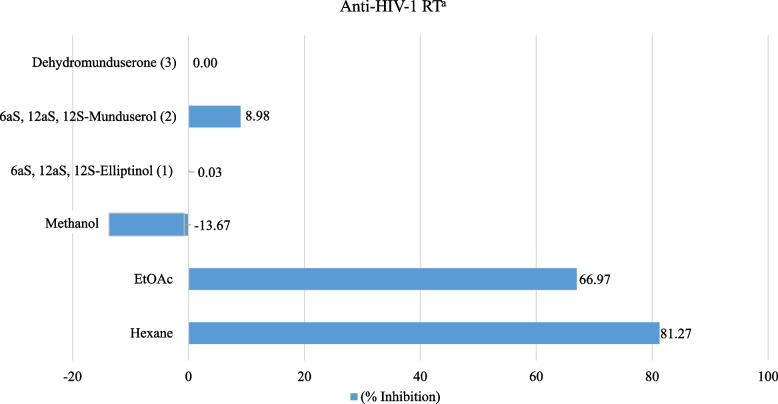
Anti-HIV-1 RT of crude extracts and pure compounds from leaves and twigs of *M. Pyrrhocarpa. *^*a*^Anti*-*HIV-1 RT activity express as %inhibition at 200 µg*/*mL: very active (VA) =  > 70% inhibition, moderately active (MA) = 50% to 69% inhibition, weakly active (WA) = 30% to 50% inhibition and inactive (I) =  < 30% inhibition; For determination of IC_50_ in the HIV-1 RT assay, the coefficients of determination, R^2^, were 0.98–0.99 in all assays for 50% end point

**Fig. 4:**
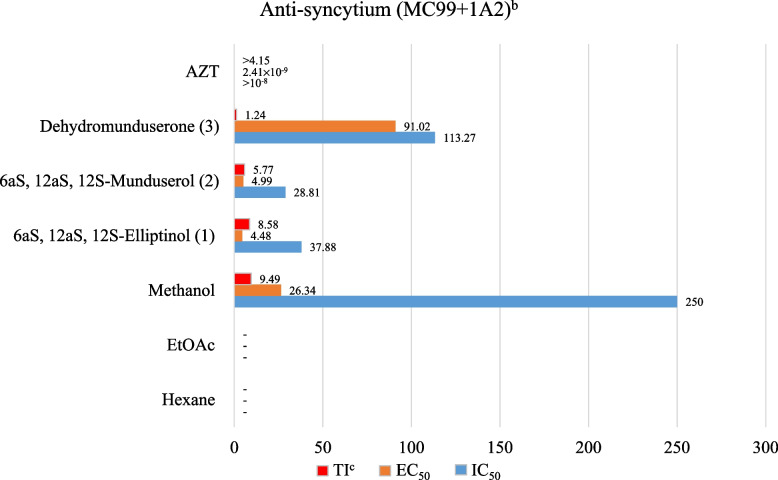
Anti*-*syncytium MC99 + 1A2 of crude extracts and pure compounds from leaves and twigs of *M. Pyrrhocarpa*. ^*b*^Anti-syncytium *(*MC99 + 1A2*)* EC_50_ = dose of compound that reduced 50% syncytium formation by ΔTat/RevMC99 virus in 1A2 cells. AZT, averaged from three experiments, EC_50_ 3.95 × 10^− 3^ μM;.^*c*^TI, Therapeutic Index: IC_50_/EC_50_

The hexane and EtOAc extracts showed the highest %inhibition of HIV-1 RT, at values of 81.27 and 66.97 at 200 µg/mL, while the MeOH extract was inactive. Compounds (1) and (2) reduced syncytium formation in 1A2 cells at EC_50_ values of 4.48 and 4.99 μM, respectively. AZT was used as a positive control, as shown in Figs. 3 and 4.

### Cytotoxicity activity

**Fig. 5:**
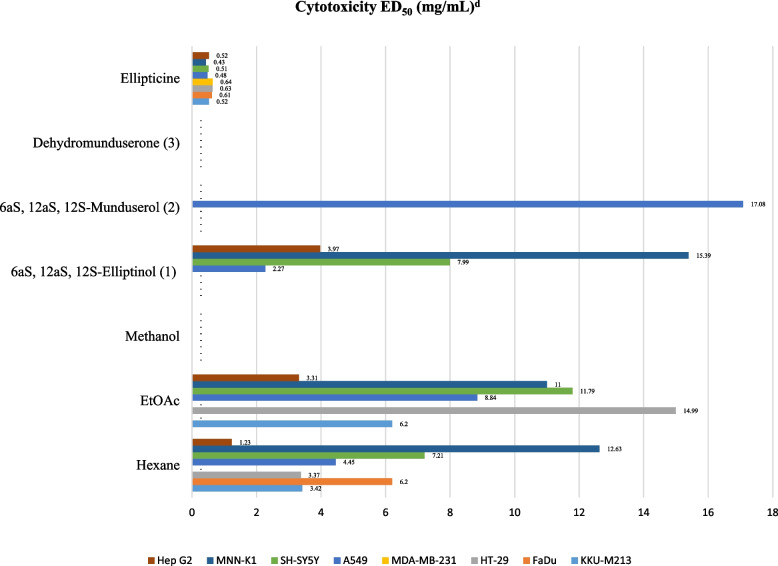
Cytotoxicity study of crude extracts and isolated compounds from leaves and twigs of *M. Pyrrhocarpa*. ^d^Cytotoxic assay: ED_50_ less than 20 µg/mL were considered active for extracts and ED_50_ less than 4 µg/mL were considered active for pure compounds. Cancer cell lines: KKU-M213 *(*Human intrahepatic cholangiocarcinoma*)* FaDu *(*Human squamous cell carcinoma*)* HT-29 *(*Human colon adenocarcinoma*)* MDA-MB-231 *(*Human mammary gland/breast adenocarcinoma*)* A 549 *(*Human lung adenocarcinoma*)* SH-SY5Y *(*Human neuroblastoma*)* MNN-K1 *(*highly differentiated immortalized human cholangiocyte cell line*)* Hep G2 *(*Human hepatocellular carcinoma*)*

The hexane extract showed cytotoxicity against KKU-M213, FaDu, HT-29, A549, SH-SY5Y, MNN-K1, and Hep G2 cells at ED_50_ values of 3.42, 6.20, 3.37, 4.45, 7.21, 12.63, and 1.23 µg/mL, respectively, while the ethyl acetate extract showed cytotoxicity against KKU-M213, HT-29, A549, SH-SY5Y, MNN-K1, and Hep G2 cells at ED_50_ values of 6.20, 14.99, 8.84, 11.79, 11.00 and 3.31 µg/mL, respectively. 6a*S*, 12a*S*, 12*S*-elliptinol (1) showed cytotoxicity against A549 and HepG2 at ED_50_ values of 2.27 and 3.97 µg/mL. Ellipticine [41, 42] was used as a positive control, as shown in Fig. 5.

## Discussion

### Structure elucidation

**Fig. 6:**
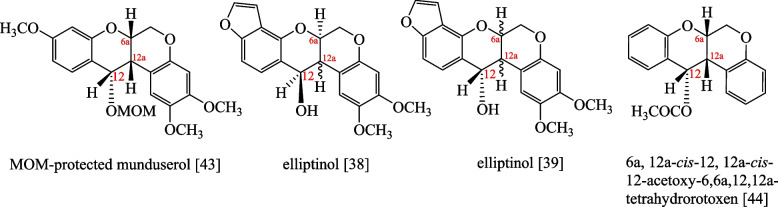
Structures of MOM-protected munduserol [43], elliptinol [38, 39], and 6a, 12a-*cis*-12, 12a-*cis*-12-acetoxy-6,6a, 12, 12a-tetrahydrorotoxen [44]

Although the spectral data of compounds (1) and (2) were consistent with previous reports [38–40], it was noted that the coupling constants and proton orientation of compounds (1) and (2) at positions 6a, 12a, and 12 of MOM-protected munduserol [43], elliptinol [38, 39], 6a, 12a-*cis*-12, 12a-*cis*-12-acetoxy-6,6a,12,12a-tetrahydrorotoxen [44], compounds (1), and (2) are quite different, and the structures and ^1^H-NMR data of the four compounds are shown in Fig. 6 and Table 2.

**Table 2:** Comparison of chemical shift and coupling constant values of MOM-protected munduserol [43], elliptinol [38, 39], 6a, 12a-*cis*-12, 12a-*cis*-12-acetoxy-6,6a,12,12a-tetrahydrorotoxen [44], compound (1) and (2)

**Position**	**MOM-protected munduserol** [43] **(*J* in Hz)**	**Elliptinol** [38] **(*J* in Hz)**	**Elliptinol** [39] **(*J* in Hz)**	**6a, 12a-*cis*-12, 12a-*cis*-12-acetoxy-6,6a,12,12a-Tetrahydrorotoxen **[44]** (*J* in Hz)**	**Compound (1) (*J* in Hz)**	**Compound (2) (*J* in Hz)**
6a	4.84, d, (3.3)	5.00, br.ddd	4.94, m	4.92, dd, (5.1, 5.2 Hz)	4.33, dt (10.2, 4.3)	4.16, td, (4.3, 10.0)
12a	3.96, s	3.65, br.dd	3.48 t, (4.8)	3.65, dd, (5.1, 5.3)	3.15, t (10.2)	3.04, t, 10.0)
12	5.05, s	6.44, br.d	5.04, d, (4.8)	6.38, d, (5.3)	4.98, t (10.2)	4.74, d, (10.0)

**Fig. 7:**
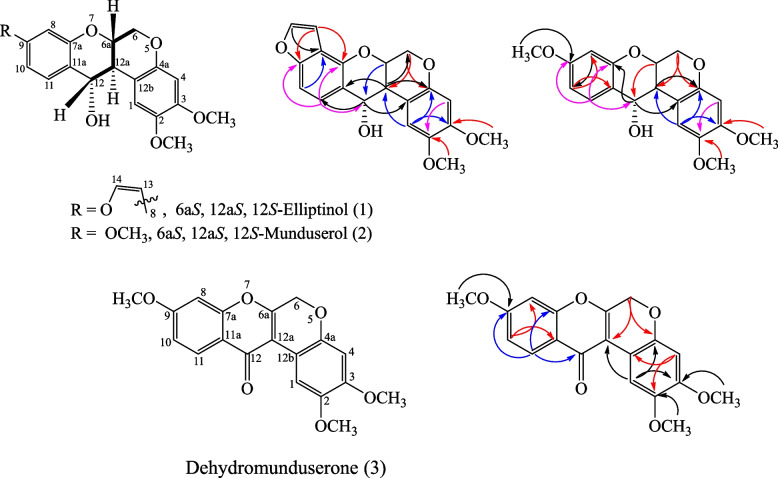
Key COSY (  ) and HMBC (  ) correlations of 6a*S*, 12a*S*, 12*S*-elliptinol (1), 6a*S*, 12a*S*, 12*S*-munduserol (2) and dehydromunduserone (3)

**Fig. 8:**
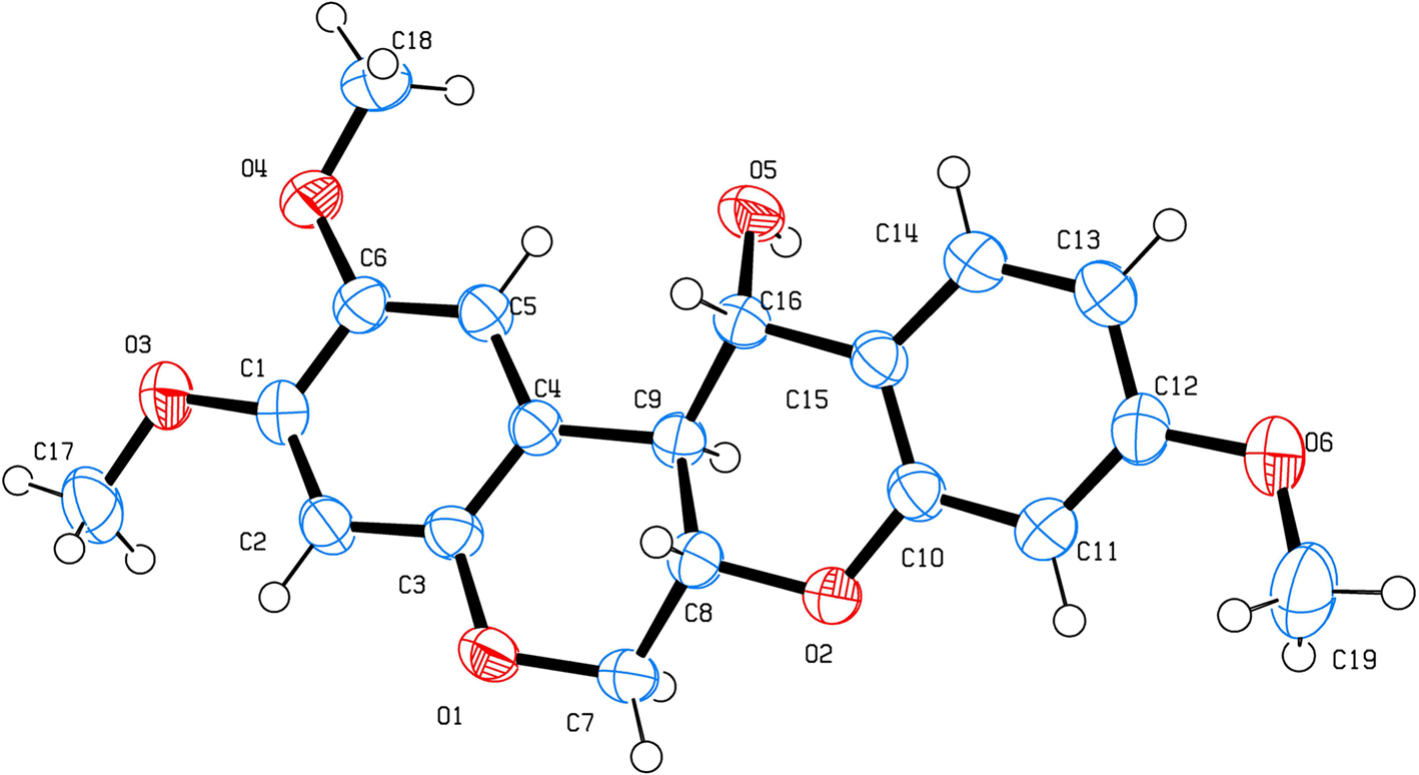
X-ray crystal structure of 6a*S*, 12a*S*, 12*S*-munduserol (2)

From Table 2, the coupling constant between H_12a_ and H_12_ was found to be 10.2 Hz in compound (1) and 10.0 Hz in compound (2), which suggested that H_12a_ and H_12_ in compound (1) and compound (2) were *trans* to each other. This conclusion was contradictory to the findings in previous reports [38, 39, 43, 44]. To settle this discrepancy, compound (2) was subjected to X-ray analysis, which confirmed that H_6a_, H_12a_ and H_12_ were in fact H_6aβ_, H_12aα_, and H_12β,_ respectively. The absolute configuration of compound (2) was therefore 6a*S*, 12a*S*, 12*S*. Comparison of the ^1^H-NMR data of compound (1) with those of 6a*S*, 12a*S*, 12*S*-munduserol (2) led to the conclusion that compound (1) was 6a*S*, 12a*S*, 12*S*-elliptinol (1). In this work, 6a*S*, 12a*S*, 12*S*-elliptinol (1) and 6a*S*, 12a*S*, 12*S*-munduserol (2) from the leaves and twigs of *M. pyrrhocarpa* afforded useful spectroscopic and XRD data, making it possible to assign the protons at the 6a, 12a, and 12 positions. The key COSY (  ) and HMBC (  ) correlations of compounds (1–3) are shown in Fig. 7, and the X-ray crystal structure of compound (2) is shown in Fig. 8.

## Bioactivities

Regarding antibacterial activity, the crude EtOAc extract from *M. pyrrhocarpa* showed good antibacterial activity against *E. Coli* and *S. flexneri* at MIC and MBC values ​​of 6.25 mg/mL. Compounds (1–3) inhibited 9 strains of bacterial cells at MIC and MBC values of 3 and > 3 mg/mL, which was more prominent than other *Milletia* genera tested in previous reports [45, 46]. In anti-HIV activity, compounds (1) and (2) from M. *pyrrhocarpa* showed anti-syncytium activity with better IC_50_ and EC_50_ values than that of the pure compound found in *Ventilago harmandiana* [47]. In in vitro cytotoxic activity, crude hexane, ethyl acetate, and compound (1) from *M. pyrrhocarpa* showed cytotoxicity against A549 (human lung adenocarcinoma) cells with better ED_50_ values at 4.45, 8.84, and 2.27 µg/mL than those of the crude extract and pure compounds from *Garcinia speciosa* Wall [48]. In addition, compared to phenylacetylshikonin analogues, compound (1) showed comparable and better cytotoxicity to A549 cells [49]. Extracts and purified compounds (1–3) from *M. pyrrhocarpa* showed bioactivity, especially compound (1), which showed potent anticancer activity. This work does possess inherent limitations. The scarcity of these compounds makes it impossible to test other biological activities, such as anti-inflammatory and antidiabetic activities, and perform in vivo studies. Further molecular modelling and computational studies, such as molecular docking and molecular dynamics techniques, are desirable. Through these studies, the interaction between the tested compounds and cells could be predicted to obtain suitable structures so that appropriate syntheses can be carried out.

## Conclusion

This is the first phytochemical investigation of the leaves and twigs of *M. pyrrhocarpa* and the first study to examine their biological activity. Interestingly, previous reports did not establish the absolute configuration of rotenol (1) and (2) [38–40]. This report established the complete structure, spectral data of compounds (1) and (2), and X-ray data of (2). Furthermore, bioassays such as antibacterial, anti-HIV virus, and cytotoxicity on rotenol (1), (2), and rotenone (3) provided significant data that could be used for further study.

## Supplementary Information

**Additional file 1.**

## Data Availability

The datasets generated and analysed during the current study are available in the corresponding author and the Crystallographic Open Database (COD) number 3000415 repository, [http://www.crystallography.net/cod/result.php].
