# Supplementary material for: Study on the absolute configuration and biological activity of rotenoids from the leaves and twigs of Millettia pyrrhocarpa Mattapha, Forest & Hawkins, sp. Nov
Source: BMC Complement Med Ther. 2023 May 4;23:147. doi: 10.1186/s12906-023-03963-4 (PMC10161675; doi:10.1186/s12906-023-03963-4)
Supplement: Supplementary file 1 — Additional file 1. [file 12906_2023_3963_MOESM1_ESM.docx]

**Additional file 1**

**Study on the absolute configuration and biological activity of rotenoids from the leaves and twigs of *Millettia pyrrhocarpa* Mattapha, Forest & Hawkins, sp. Nov**

Suda Sanunbunudom^1^, Atchara Kaewnoi^2^, Wilart Pompimon^1^, Samroeng Narakaew^1^, Suwadee Jiajaroen^3^, Kittipong Chainok^3^, Narong nuntasaen^4^, Kanoknetr Suksen^5^ , Arthit Chairoungdua^5,6,7^, Jitra Limthongkul^8^ Chanita Naparswad^8^, Suttiporn Pikulthong^9^, Puttinan Meepowpan^10^, Boonthawan Wingwon^11^, Nichapa Charoenphakinrattana^12^, and Phansuang Udomputtimekakul^1*^

^1^Department of Chemistry, Faculty of Science and Center of Innovation in Chemistry, Lampang Rajabhat University, 52100, Lampang, Thailand

^2^Department of Thai Traditionnal Medicine, Faculty of Science and Technology, Bansomdejchaopraya Rajabhat University, 10600, Bangkok, Thailand

^3^Thammasat University Research Unit in Multifunctional Crystalline Materials and Applications (TUMcMa), Faculty of Science and Technology, Thammasat University, 12121, Pathum Thani, Thailand

^4^ Department of Chemistry, Faculty of Science and Center of Innovation in Chemistry, Mahidol University, Rama VI Road, Bangkok 10400, Thailand

^5^Department of Physiology, Faculty of Science, Mahidol University, Rama 6 Road, Bangkok, 10600, Thailand

^6^Excellent Center for Drug Discovery (ECDD), Mahidol University, Bangkok, 10600, Thailand

^7^Toxicology Graduate Program, Faculty of Science, Mahidol University, Bangkok, 10600, Thailand

^8^Department of Microbiology, Faculty of Science, Mahidol University, Bangkok, 10600, Thailand

^9^Department of Chemistry, Faculty of Science, Mahidol University, Bangkok, 10600, Thailand

^10^Department of Chemistry, and Center for Innovation in Chemistry, Faculty of Science, Chiang Mai University, Chiang Mai 50300, Thailand

^11^Department of Management Science, Faculty of Management Science, Lampang Rajabhat University, 52100, Lampang, Thailand

^12^Life Group International, Khaochangum Subdistrict, Photharam District, 70120, Ratchaburi, Thailand

*Corresponding author: Phansuang Udomputtimekakul

phansuang@g.lpru.ac.th

Tel: +6681614

6955

1. **NMR, MS, IR, UV and optical rotation data of 6a*S*, 12a*S*, 12*S*-elliptinol** (**1**)

Figure S1. ^1^H NMR spectrum (500 MHz) of 6a*S*, 12a*S*, 12*S*-elliptinol (**1**) in CDCl_3_


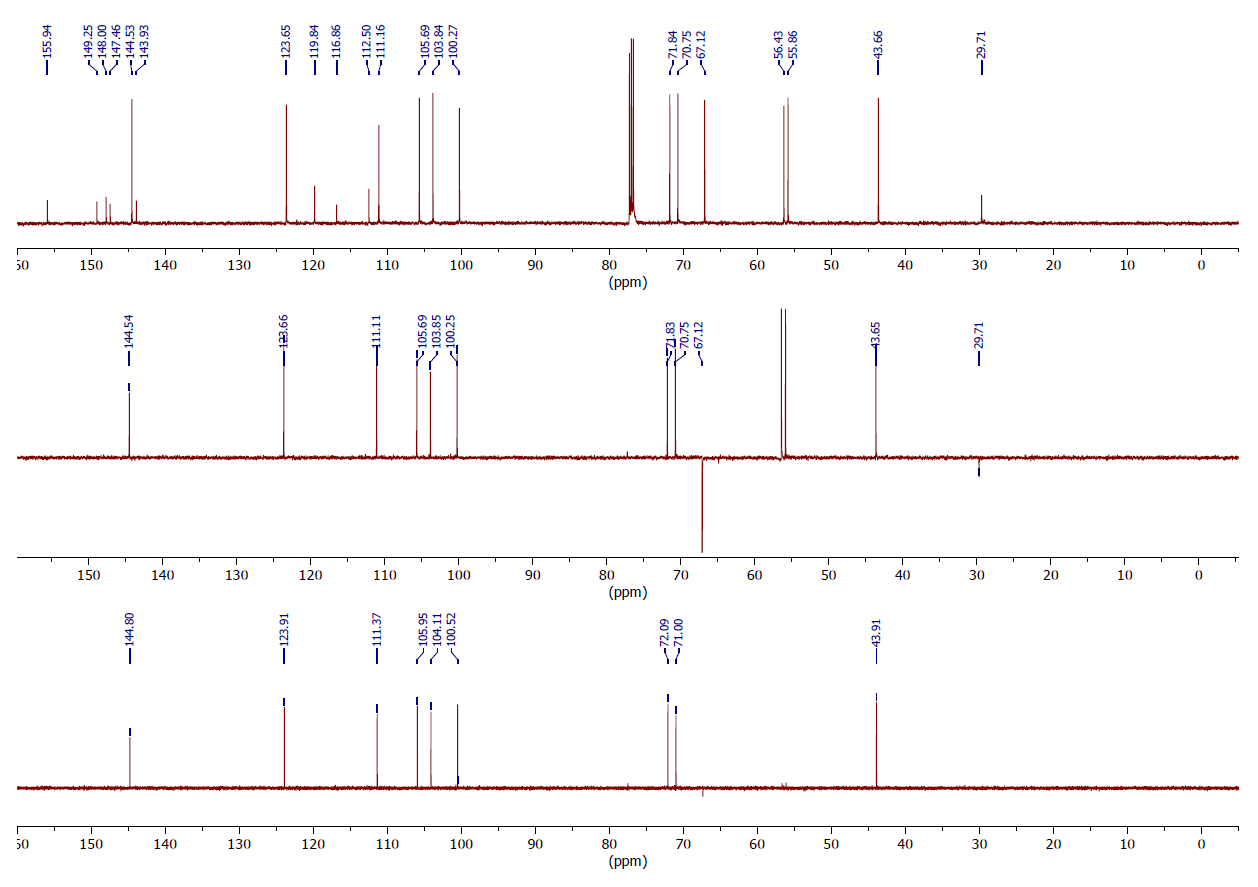


Figure S2. ^13^C NMR spectrum (125 MHz) of 6a*S*, 12a*S*, 12*S*-elliptinol (**1**) in CDCl_3_

Figure S3. ^1^H–^1^H COSY spectrum of 6a*S*, 12a*S*, 12*S*-elliptinol (**1**) in CDCl_3_


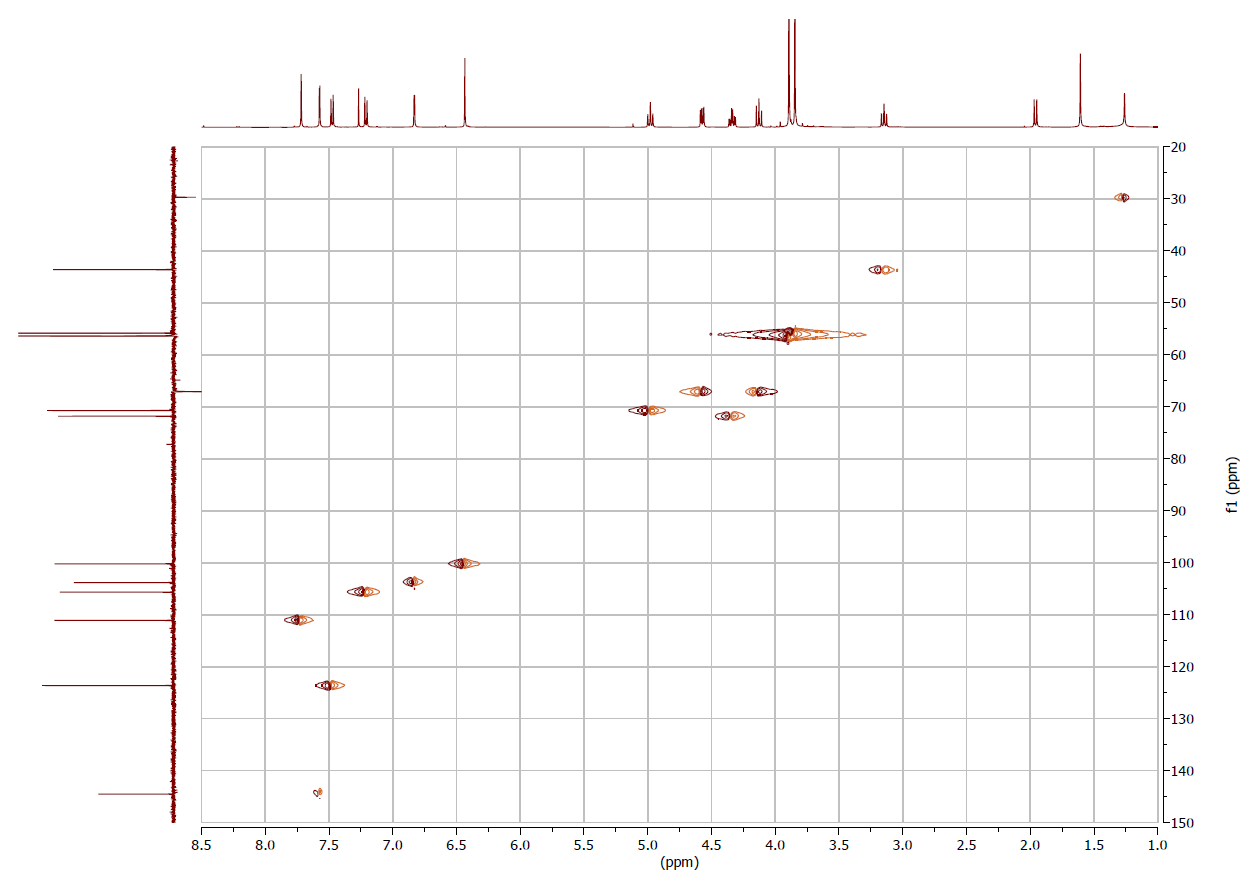


Figure S4. HSQC spectrum (125 MHz) of 6a*S*, 12a*S*, 12*S*-elliptinol (**1**) in CDCl_3_

Figure S5. HMBC spectrum (125 MHz) of 6a*S*, 12a*S*, 12*S*-elliptinol (**1**) in CDCl_3_


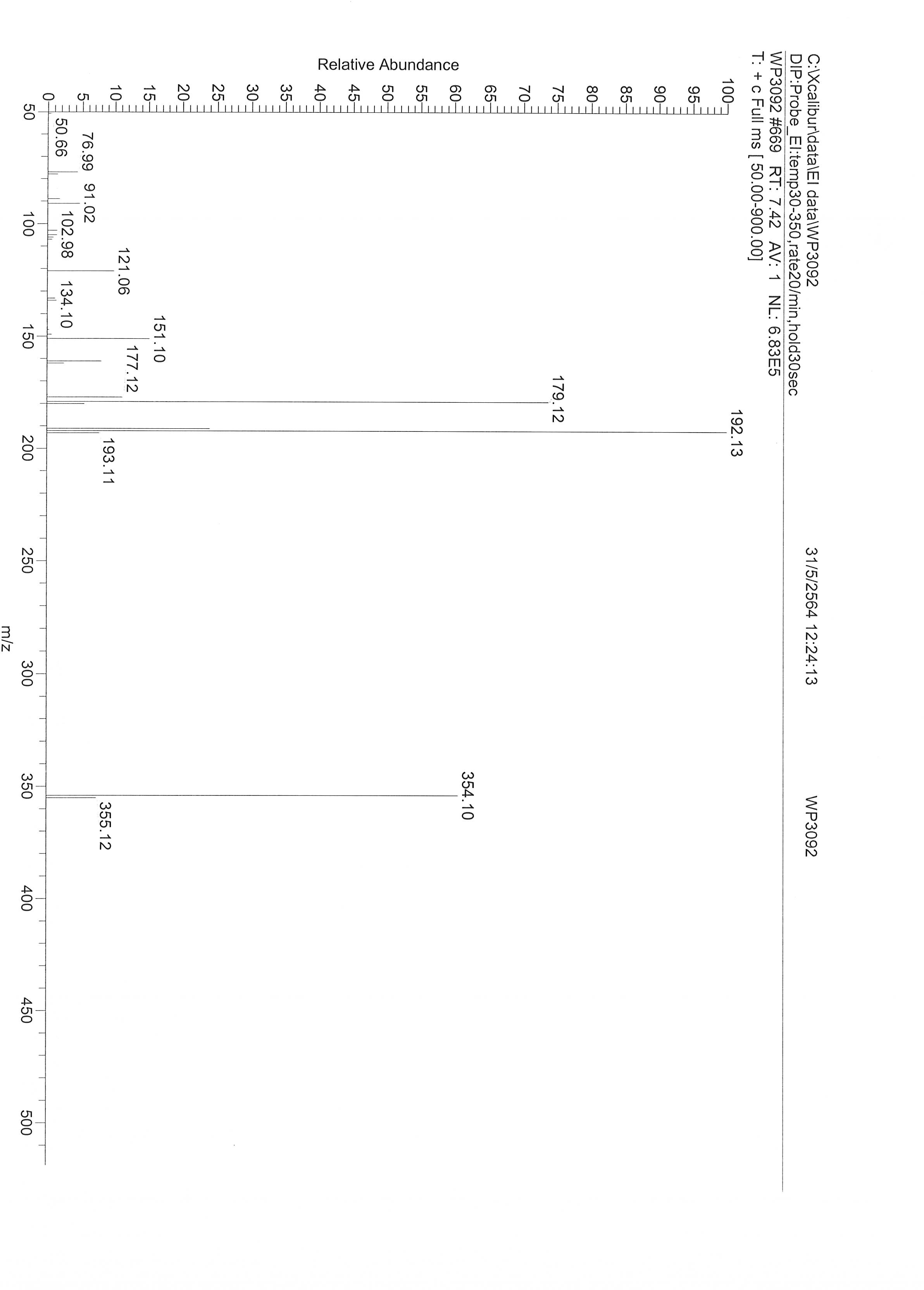

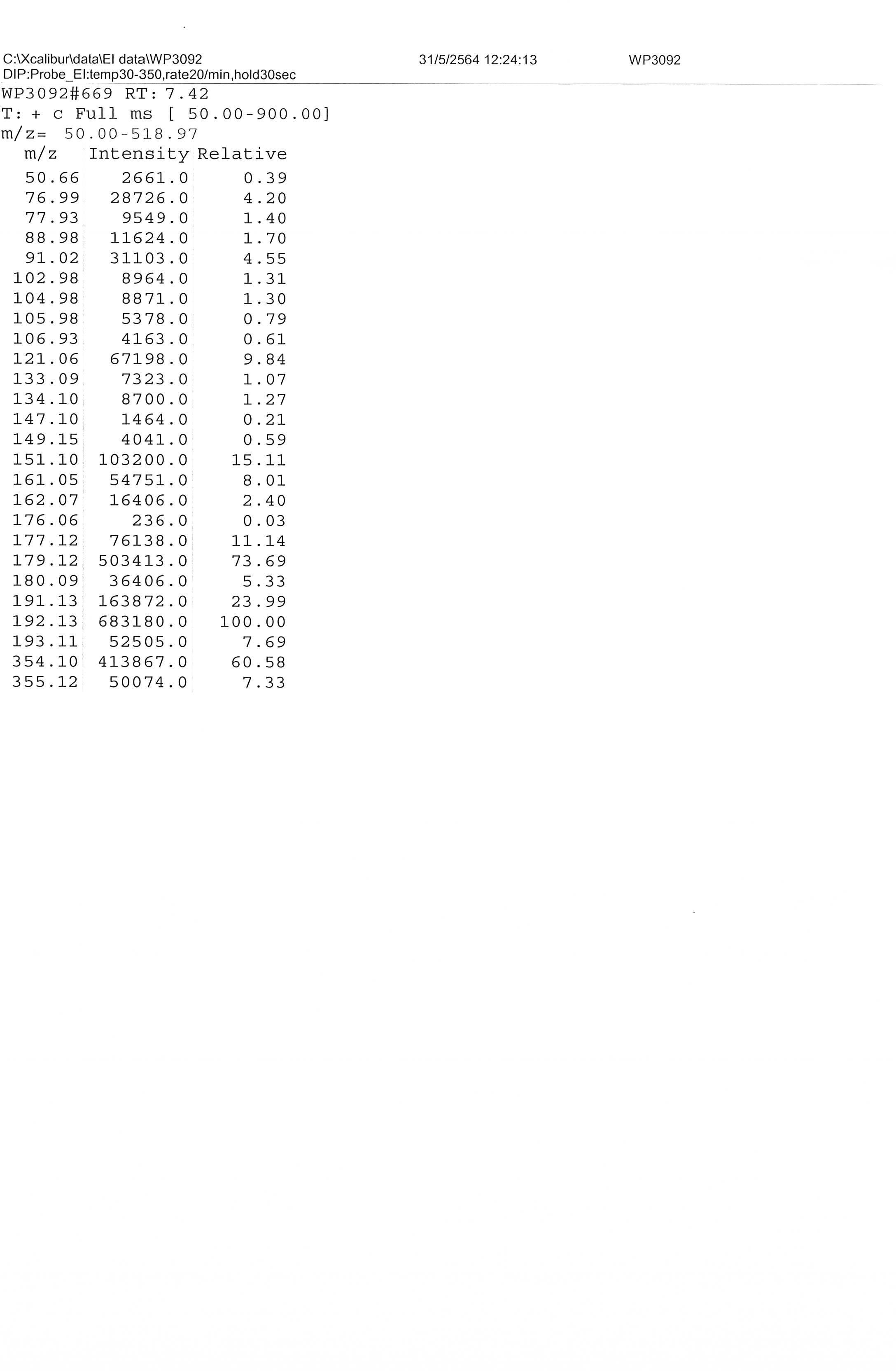


Figure S6. EI-MS spectrum of 6a*S*, 12a*S*, 12*S*-elliptinol (**1**) in CDCl_3_


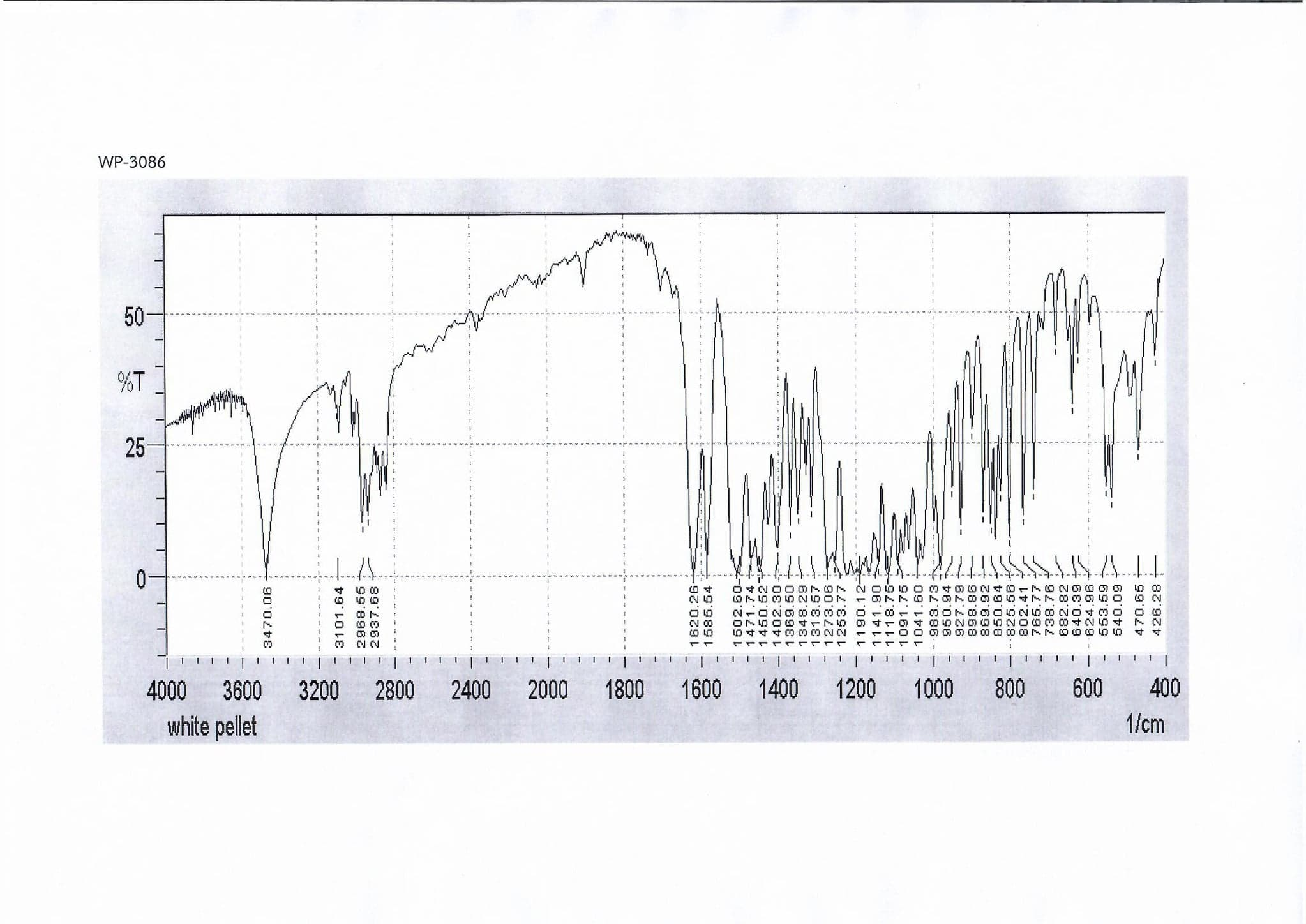


Figure S7. IR spectrum of 6a*S*, 12a*S*, 12*S*-elliptinol (**1**) in CDCl_3_

__

Figure S8. UV spectrum of 6a*S*, 12a*S*, 12*S*-elliptinol (**1**) in CDCl_3_


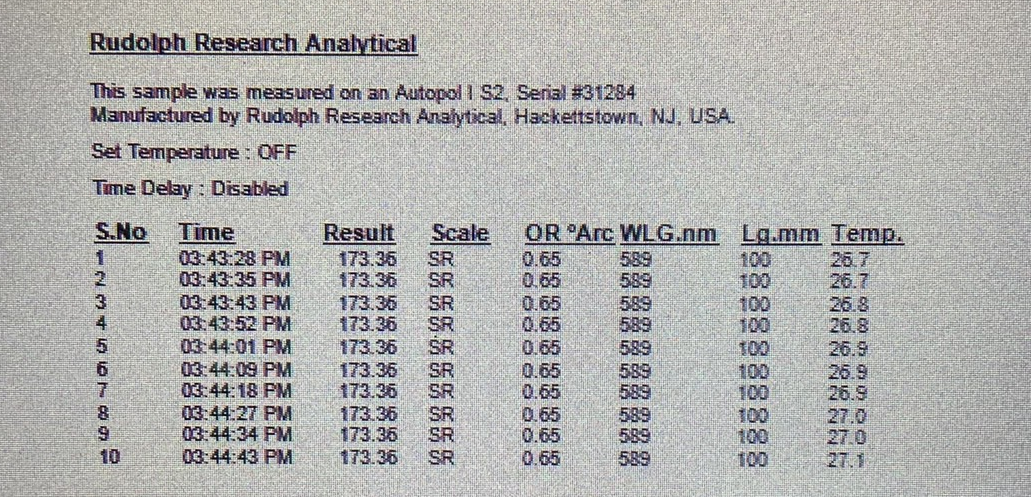


Figure S9. Optical rotation of 6a*S*, 12a*S*, 12*S*-elliptinol (**1**) in CDCl_3_

1. **NMR, MS, IR, UV and optical rotation data of 6a*S*, 12a*S*, 12*S*-munduserol (2)**

Figure S10. ^1^H NMR spectrum (500 MHz) of 6a*S*, 12a*S*, 12*S*-munduserol (**2**) in CD_3_OD


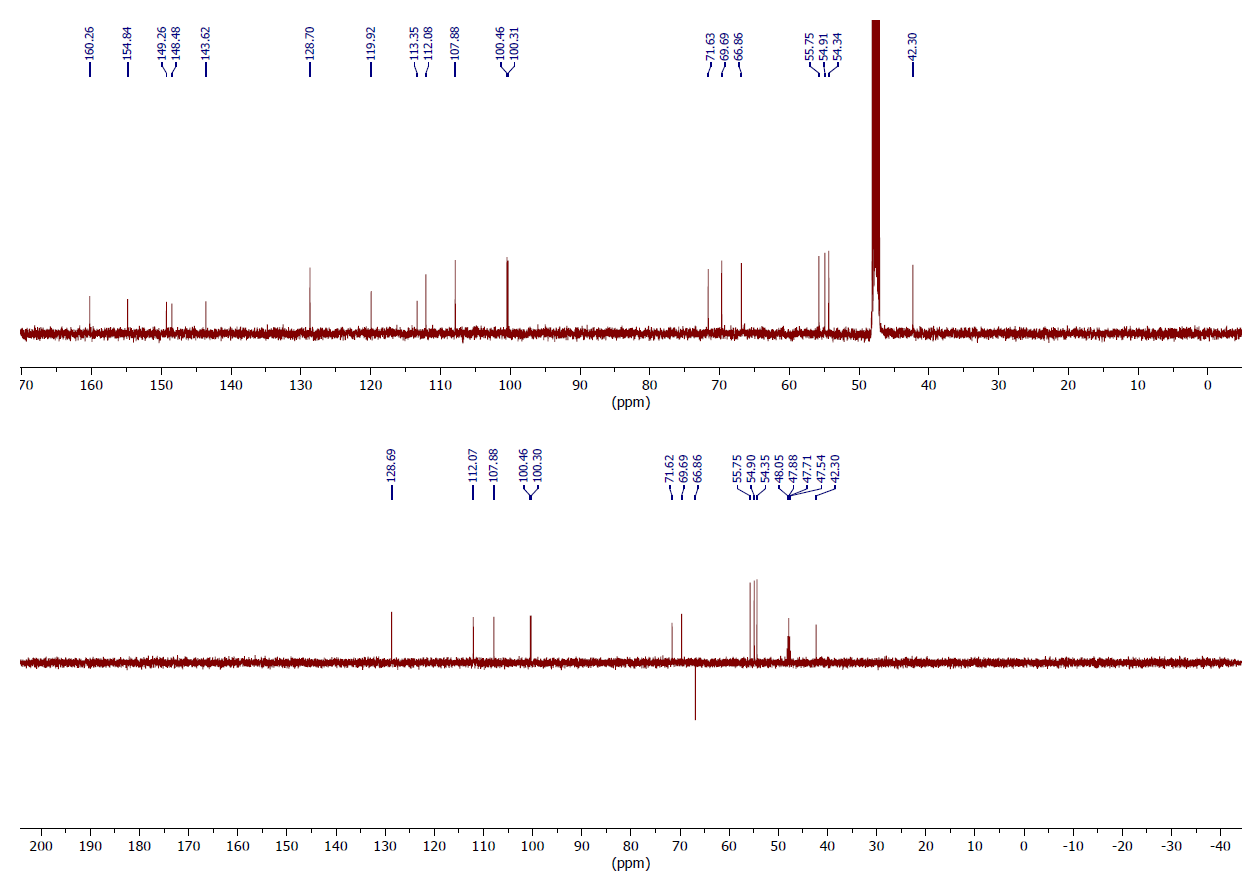


Figure S11. ^13^C NMR spectrum (125 MHz) of 6a*S*, 12a*S*, 12*S*-munduserol (**2**) in CD_3_OD


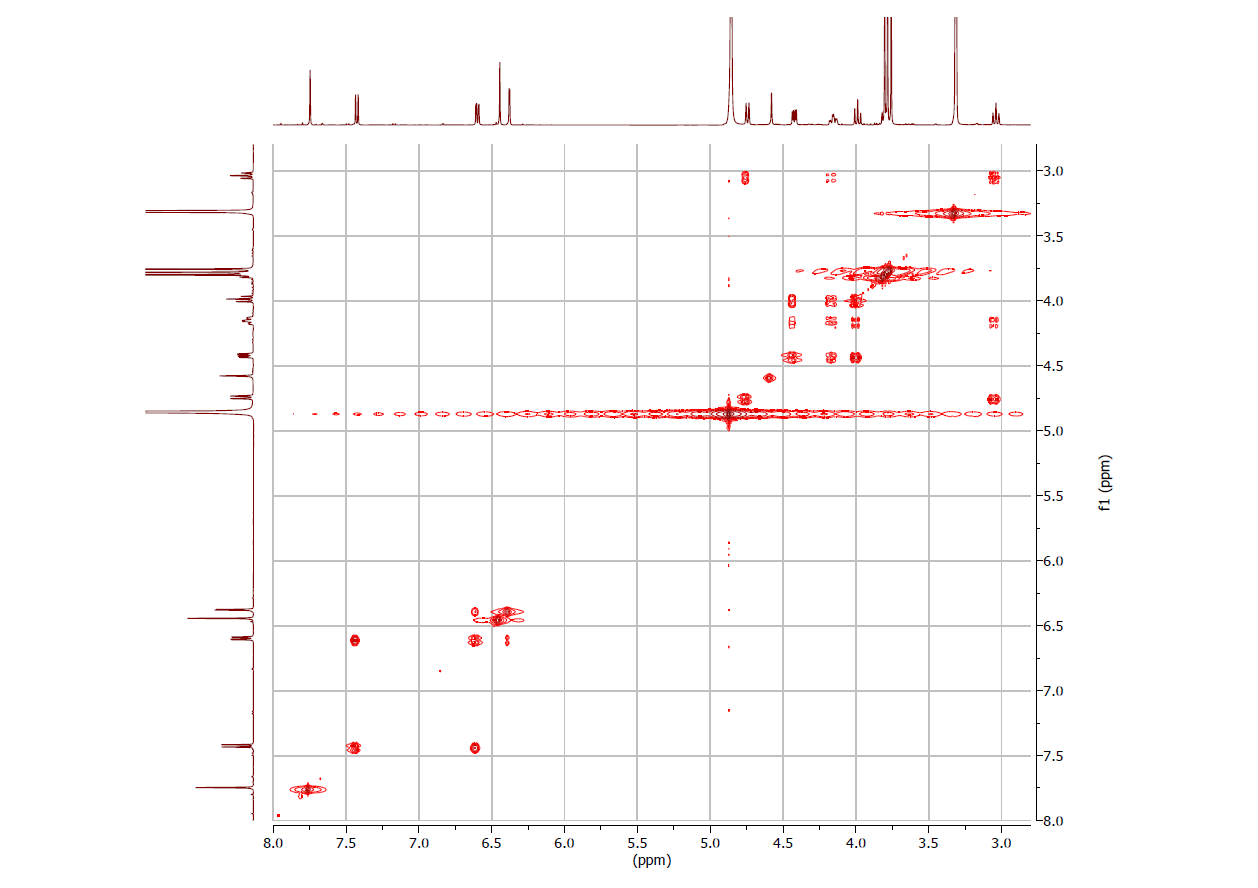


Figure S12. ^1^H–^1^H COSY spectrum of 6a*S*, 12a*S*, 12*S*-munduserol (**2**) in CD_3_OD


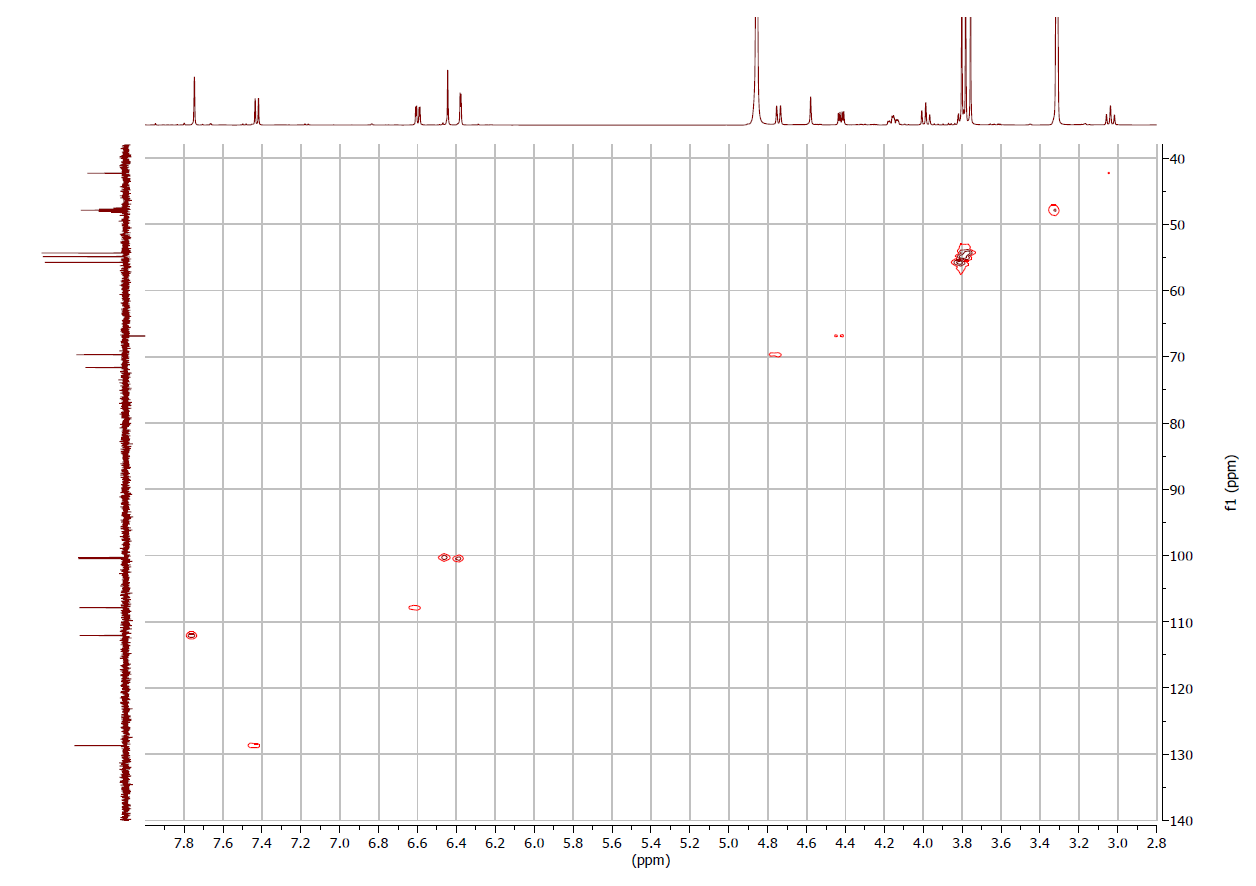


Figure S13. HSQC spectrum (125 MHz) of 6a*S*, 12a*S*, 12*S*-munduserol (**2**) in CD_3_OD


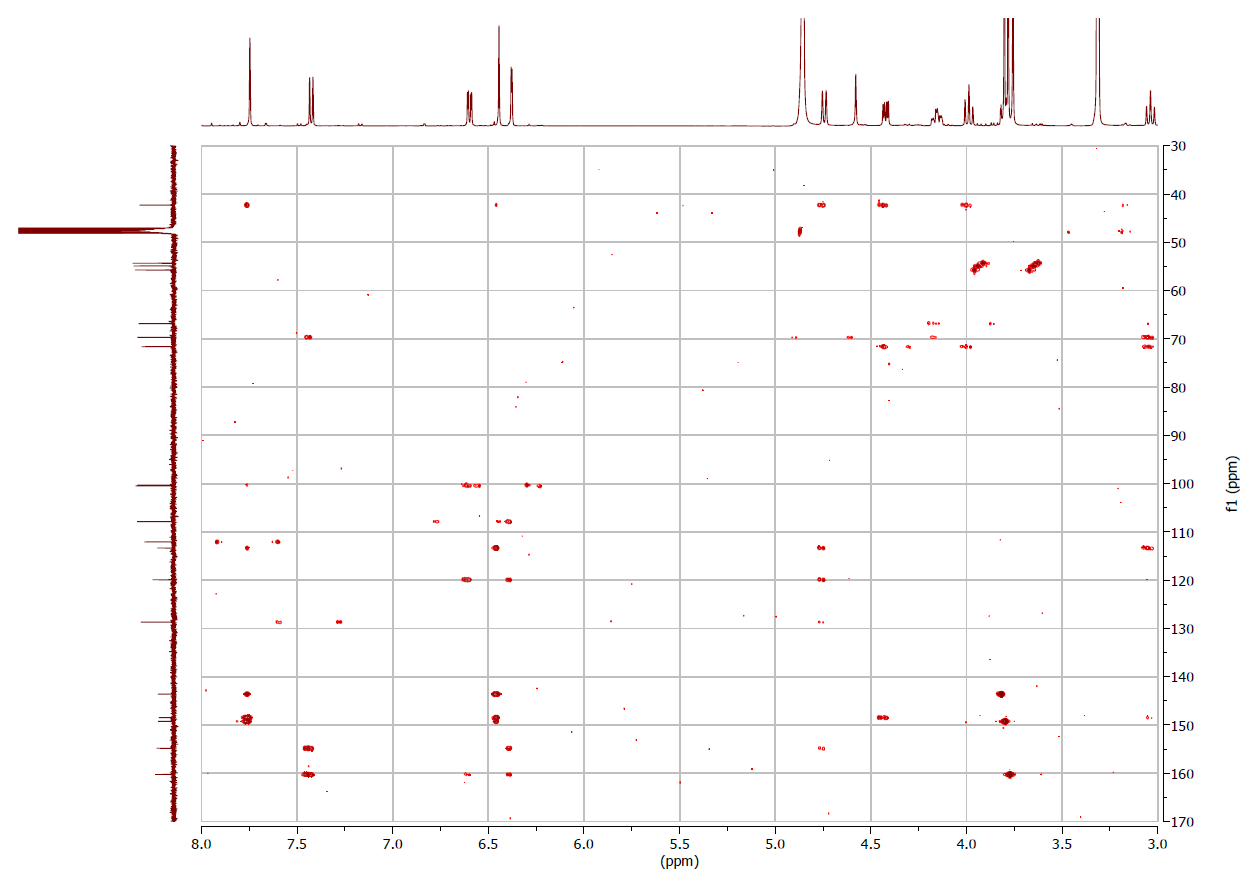


Figure S14. HMBC spectrum (125 MHz) of 6a*S*, 12a*S*, 12*S*-munduserol (**2**) in CD_3_OD

Figure S15. EI-MS spectrum of 6a*S*, 12a*S*, 12*S*-munduserol (**2**)

Figure S16. IR spectrum of 6a*S*, 12a*S*, 12*S*-munduserol (**2**)

Figure S17. UV spectrum of 6a*S*, 12a*S*, 12*S*-munduserol (**2**) in CH_3_OH

_
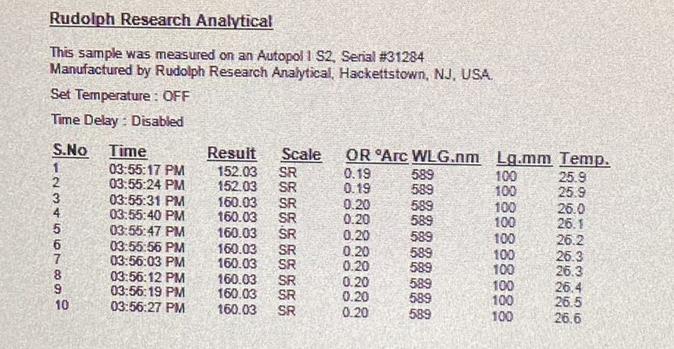
_

Figure S18. Optical rotation of 6a*S*, 12a*S*, 12*S*-munduserol (**2**) in CH_3_OH

1. **NMR, MS, IR and UV data of dehydromunduserone (3)**

Figure S19. ^1^H NMR spectrum (500 MHz) of dehydromunduserone (**3**) in CDCl_3_


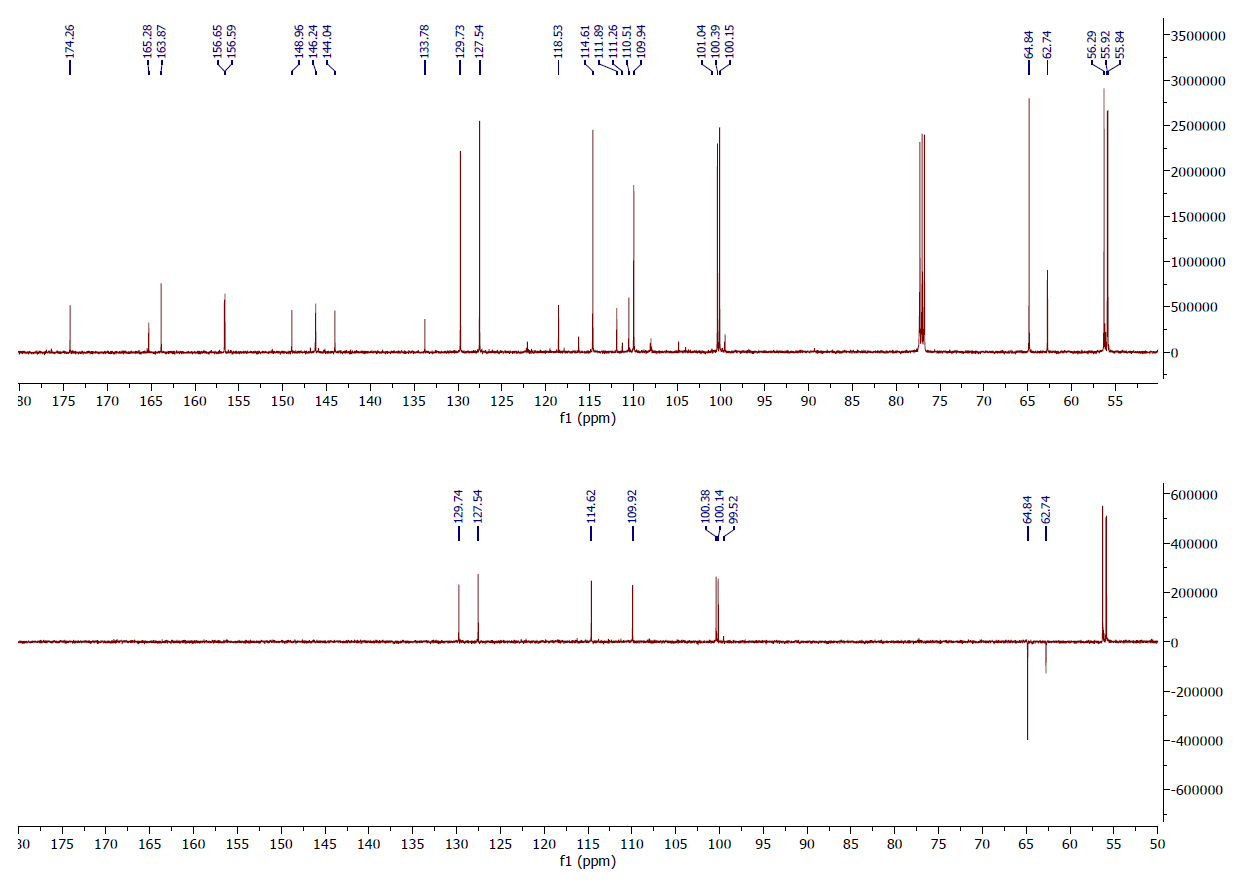


Figure S20. ^13^C NMR spectrum (125 MHz) of dehydromunduserone (**3**) in CDCl_3_

Figure S21. ^1^H–^1^H COSY spectrum of dehydromunduserone (**3**) in CDCl_3_


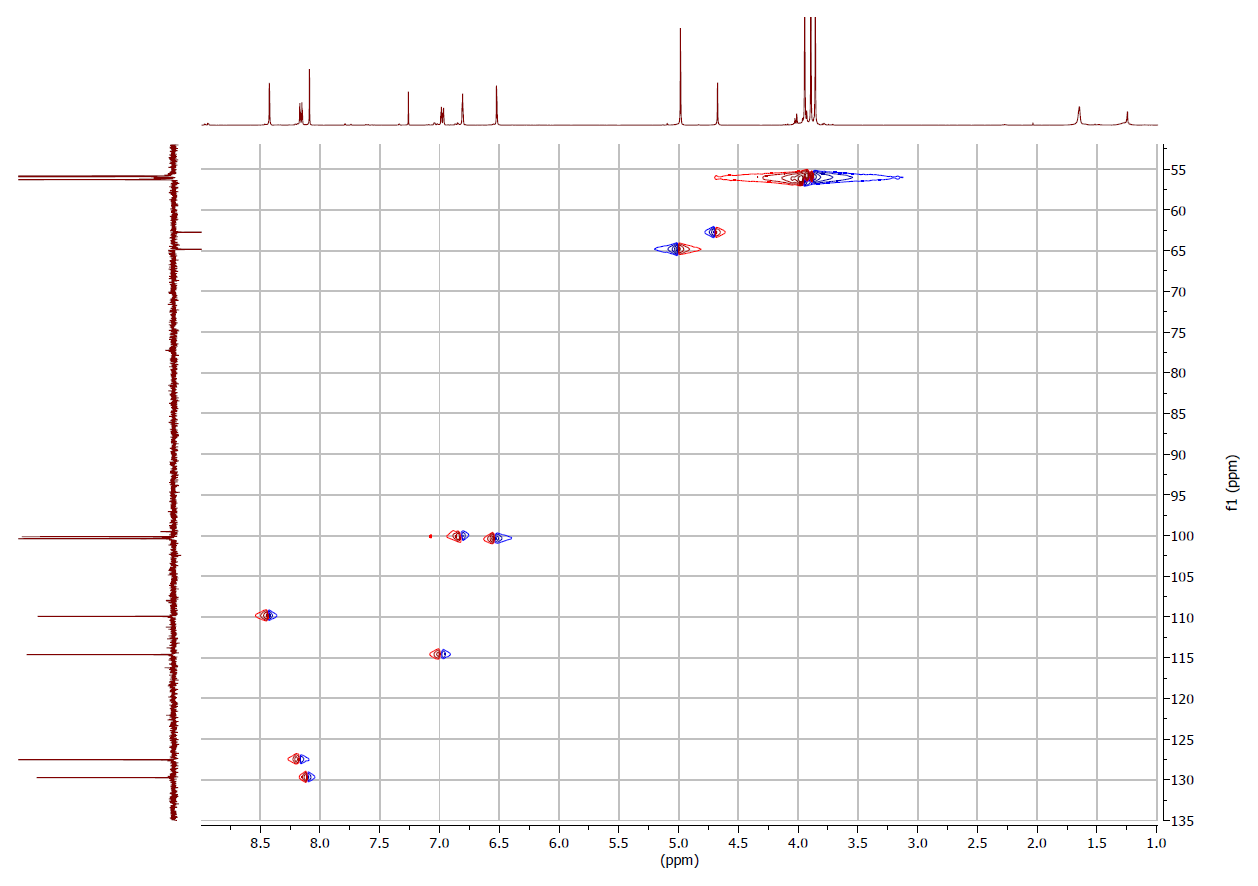


Figure S22. HSQC spectrum of dehydromunduserone (**3**) in CDCl_3_

Figure S23. HMBC spectrum of dehydromunduserone (**3**) in CDCl_3_

__

Figure S24. EI-MS spectrum of dehydromunduserone (**3**)

Figure S25. IR spectrum of dehydromunduserone (**3**)

Figure S26. UV spectrum of dehydromunduserone (**3**)
